# Recurrent Solitary Fibrous Tumor (Intracranial Hemangiopericytoma) Treated With a Novel Combined-Modality Radiosurgery Technique: A Case Report and Review of the Literature

**DOI:** 10.3389/fonc.2022.907324

**Published:** 2022-05-26

**Authors:** Alexander J. Allen, Dominic Angelo Labella, K. Martin Richardson, Jason P. Sheehan, Charles R. Kersh

**Affiliations:** ^1^ Chesapeake Regional, Riverside & University of Virginia Radiosurgery Center, Riverside Regional Medical Center, Newport News, VA, United States; ^2^ Department of Neurological Surgery, University of Virginia Health System, Charlottesville, VA, United States

**Keywords:** gamma knife (GK), solitary fibrous tumor (SFT), intracranial hemangiopericytoma, stereotactic radiosurgery (SRS), recurred cancer

## Abstract

Solitary Fibrous Tumor (SFT) is a rare and aggressive mesenchymal malignancy of the dura with a predilection for recurrence after treatment. We report a case of a SFT initially treated with subtotal surgical resection followed by a combination of Gamma Knife (GK) and linear accelerator-based radiosurgery. Forty-four days post-resection, the tumor had demonstrated radiographic evidence of recurrent disease within the post-operative bed. GK radiosurgery treatment was delivered in a “four-matrix” fashion targeting the entire surgical cavity as well as three nodular areas within this wide field. This treatment was delivered in one fraction with a stereotactic head frame for immobilization. A consolidation radiosurgery treatment course was then delivered over three additional fractions to the resection bed using a linear accelerator and mesh mask for immobilization. The total biologically effective dose (BED) was calculated as 32.50 Gy to the surgical bed and approximately 76.50 Gy to each nodular area. Almost three years post-operatively, the patient is alive and without radiographic or clinical evidence of disease recurrence. To our knowledge, no prior experiences have documented treatment of SFT using a mixed-modality, multi-fraction radiosurgery technique like the method detailed in this report. Our experience describes a combined modality, multi-fraction radiosurgery approach to treating recurrent SFT that maximizes radiation dose to the targets while minimizing complication risk. We believe this novel radiosurgery method should be considered in cases of grade II SFT post-resection.

## Introduction

Solitary fibrous tumor (SFT), known as hemangiopericytoma prior to the updated 2016 World Health Organization (WHO) guidelines, is a rare intracranial malignancy thought to originate from pericyte cells lining the capillary walls. These tumors constitute 2.5% of meningeal tumors and less than 1% of intracranial tumors (1, 2). They are usually attached to the dura, and are thus frequently mistaken for meningiomas on imaging. Unlike meningiomas, SFT are malignant and have a propensity for local recurrence and extracranial metastasis after resection (3). SFT are classified as WHO grade I or II neoplasms with some anaplastic variants classified as grade III (high grade) (4). The mean age of occurrence is 43 years and men are slightly more often affected than women (male/female ratio 1.4:1) (2).

**Figure 1:**
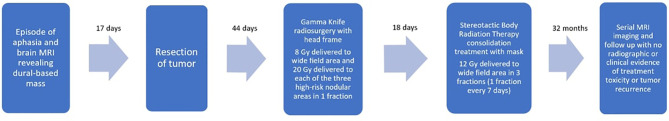
Clinical and Treatment Timeline.

While resection is the most common initial therapy for SFT, stereotactic radiosurgery (SRS), specifically Gamma Knife (GK) radiosurgery, has emerged as a promising adjuvant treatment. Multiple studies have suggested a likely survival benefit of adjuvant SRS for SFT compared to resection alone (5–7). However, published data still show high recurrence rates of SFT despite adjuvant radiosurgery, implying a need for more effective treatment plans (5). There is evidence suggesting that higher cumulative radiation dose from adjuvant GK correlates with significantly improved progression free survival (PFS) for SFT (6, 8). However, delivering higher doses of radiation can increase risk of adverse effects, and dividing treatment across multiple fractions requires multiple invasive headframe placements for most GK systems. We describe treating a case of grade II SFT post-resection using a single-fraction GK treatment with multiple treatment volumes followed by a multi-fraction consolidation radiosurgery treatment using a linear accelerator (**Figure 1**). Most of the literature documenting radiosurgery treatment for SFT focuses on single-fraction GK specifically. To our knowledge, there are no published studies documenting adjuvant SRS treatment of SFT administered in multiple fractions using two different radiotherapy delivery systems.

## Case Description

A 61-year-old male with a past medical history of hypertension initially presented to the emergency room after experiencing a sudden episode of expressive aphasia for one minute while resting at home. His symptoms had completely resolved upon arrival to the emergency department. He denied any nausea, vomiting, vision changes, or witnessed seizure-like activity. Neurological exam in the emergency department was non-focal.

Subsequent magnetic resonance imaging (MRI) of the brain revealed a 5.2 x 5.6 cm low T1, high FLAIR, high T2, homogenously enhancing dural-based mass in the posterior frontal lobe. The mass extended across the midline with invasion and transgression of the interhemispheric fissure and superior sagittal sinus with minimal surrounding vasogenic edema. After neurosurgical evaluation, dexamethasone and levetiracetam were started and the patient was scheduled for surgery. The patient underwent craniotomy and resection of the presumed meningioma 17 days after initial presentation. The tumor was dissected from within the sagittal sinus. However, the tumor was only partially resected as the visible disease could not be fully excised from the patent sinus anteriorly or posteriorly. The patient tolerated surgery without complications.

Surgical pathology was consistent with a malignant Hemangiopericytoma/Solitary Fibrous Tumor (SFT), World Health Organization (WHO) grade II/III, Ki67 2%, and diffusely *STAT6* positive. A brain MRI performed 44 days post-operatively revealed post-surgical encephalomalacia in the high right posterior region with marginal nodular enhancement along the left lateral and posterior surgical bed. These findings were compatible with recurrent disease. Due to the low likelihood of achieving a complete resection in a subsequent operation, as well as patient preference, repeat surgery was deferred in favor of radiosurgery. We sought to deliver a high cumulative dose to the resection bed based on the literature showing better outcomes in treating SFT with higher overall radiation doses from adjuvant SRS. However, due to the large size of the treatment area, we could not achieve an adequate cumulative dose in a single headframe-based Gamma Knife (GK) radiosurgery treatment without risking significant complications. The decision was thus made to pursue a hypofractionated radiotherapy regimen, with each treatment separated by at least one week. Because our institution at the time was equipped with a GK Perfexion™ system that only allowed for headframe-based treatment, we opted for a combination technique of GK followed by stereotactic body radiotherapy (SBRT) with a linear accelerator.

**Figure 2:**
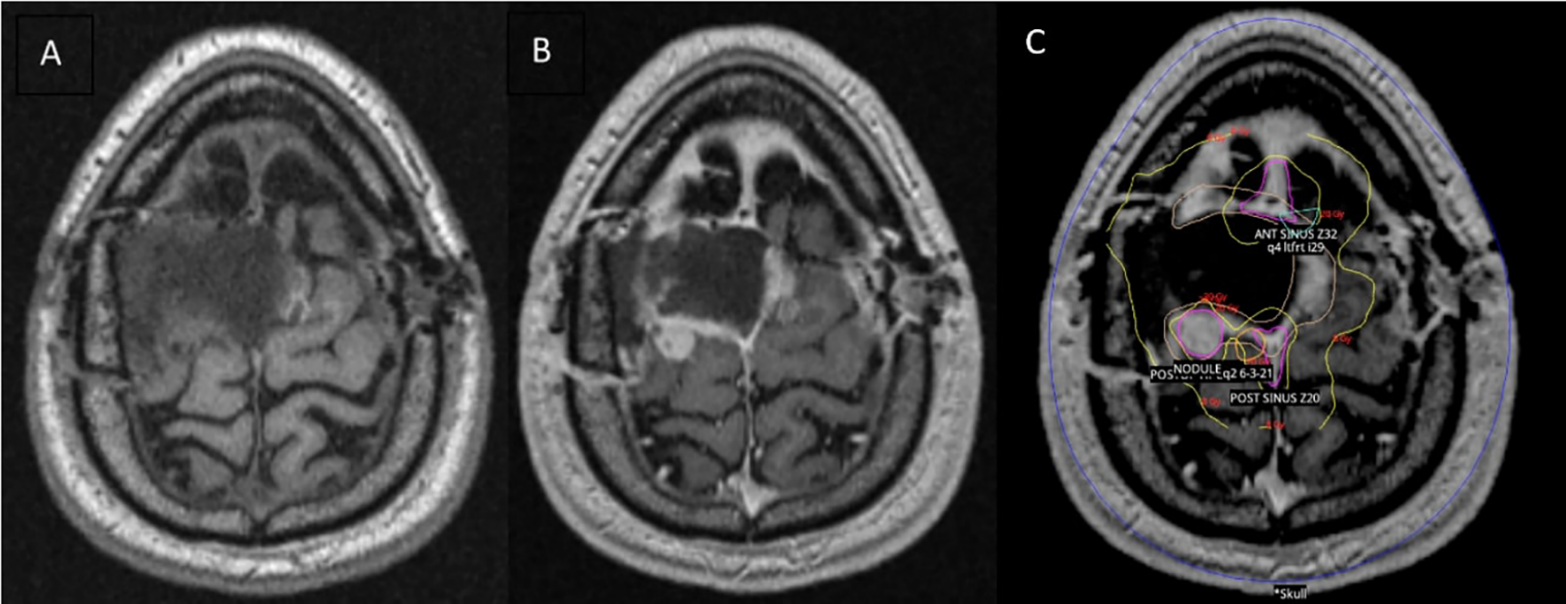
Brain MRI performed 44 days post-operatively and overlying radiotherapy treatment plan. **(A)** MRI T1 pre-contrast. **(B)** MRI T1 post-contrast showing peripheral nodular enhancement on the left lateral and posterior surgical bed. **(C)** Gamma Knife (GK) radiosurgery treatment plan overlying T1 post-contrast MRI brain. The wide post-operative area is represented by the yellow contour. The pink contours delineate the three nodular, high-risk areas.

The patient underwent GK to the tumor bed using a four-matrix paradigm: the broad, post-operative region was treated with 8 Gy to the 50% isodose line while three radiographically enhancing, nodular foci within this region were simultaneously treated with 20 Gy each to the 50% isodose line (**Figure 2**). A stereotactic head frame was attached to the calvarium prior to treatment, and the treatment was delivered in one fraction. SBRT consolidation treatment to the area was then performed with a Varian Edge™ linear accelerator and a thermoplastic mesh mask for immobilization. The treatment plan was transferred from the GammaPlan™ planning system to the ARIA™ linear accelerator planning software. The first fraction of consolidation SBRT was delivered 18 days after initial GK treatment. This consolidation plan consisted of treating the broad, post-operative area to 12 Gy in 3 fractions, one fraction every seven days. The three high-risk nodular areas were contoured, and each received 14-16 Gy during the consolidation treatment. After completion of both the GK and consolidation treatment, each high-risk area in the tumor bed had received a total dose equivalent to 34-36 Gy, corresponding to a biologically effective dose (BED) of 76.25-76.50 Gy. The field encompassing the surgical bed received a total dose of 20 Gy, corresponding to a BED of 32.50 Gy.

**Figure 3:**
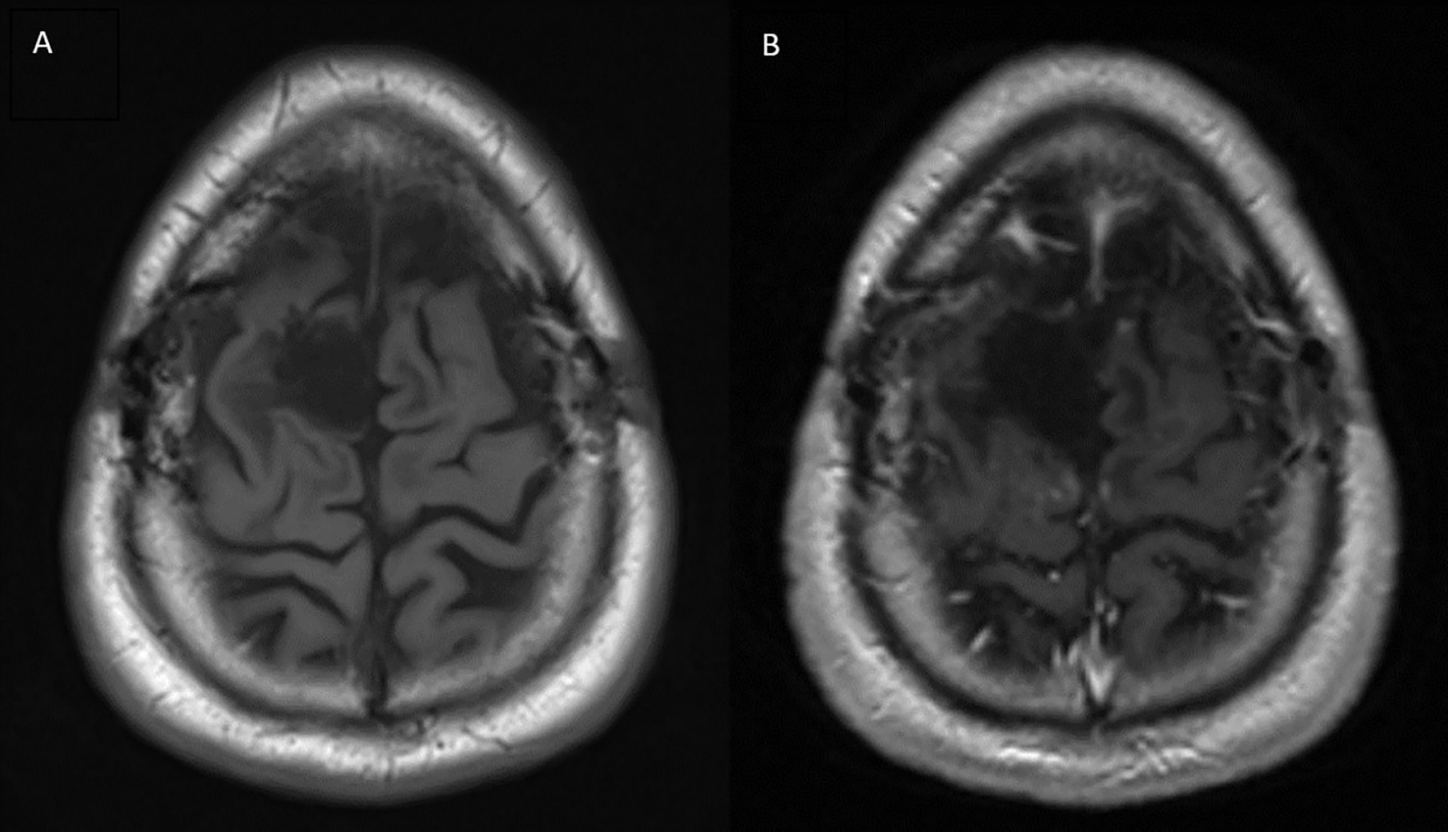
Brain MRI performed 34 months after completion of radiotherapy treatment. **(A)** T1 pre-contrast **(B)** T1 post-contrast showing no areas of peripheral or nodular enhancement.

A brain MRI three months after completion of consolidative SBRT demonstrated a subdural fluid collection and a focus of nodular dural enhancement overlying the posterior right frontal lobe measuring 0.3 x 0.7 cm. Neurosurgical follow-up at that same time revealed no clinical changes. Brain MRI and clinical follow-up roughly every 3 months thereafter demonstrated no new evidence of recurrent disease and gradual resolution of the enhancement. The most recent brain MRI performed 34 months after completing the radiosurgery treatment course showed only expected gliosis, post-surgical changes, and no enhancing foci or evidence of recurrent tumor (**Figure 3**). The patient has remained asymptomatic and without any functional deficits since completing treatment.

## Discussion

Solitary Fibrous Tumor (SFT) is a rare and aggressive intracranial mesenchymal malignancy. Prior to 2016, SFTs were defined as benign mesenchymal tumors that were clinically and pathologically distinct from hemangiopericytomas (HPCs). Recent evidence has shown that SFTs and intracranial HPCs both share the immunohistochemical features of *NAB2* and *STAT6* gene fusion as well as *STAT6* overexpression. This finding prompted the World Health Organization (WHO) to classify both conditions under the umbrella term of *solitary fibrous tumor* in their updated 2016 guidelines (9). Given the rarity of SFTs, there is no gold standard for treatment, and most evidence regarding management comes from small studies and case series. Surgical resection is the most common and most widely accepted initial treatment; however, resection alone has been shown to provide poor long-term control. While complete resections have been associated with improved survival, they are very difficult to achieve given the tendency of SFTs to infiltrate adjacent vascular structures (10). The recurrence rate of SFTs after surgery alone is estimated at 88-100% as soon as 12 months post-resection, suggesting a need for adjuvant therapy (5–7). Adjuvant chemotherapy has shown only minimal benefit, and adjuvant conventionally fractionated radiotherapy has shown mixed results in the literature (5, 6). 

There is minimal data evaluating treatment of SFT that accounts for all tumors now included under the updated 2016 WHO definition. Sung et al. identified a cohort of patients diagnosed with SFT or HPC, reclassified them according to these new guidelines, and analyzed clinical outcomes. They found that adjuvant radiotherapy (RT) was associated with improved progression-free survival, but not overall survival (OS) for grade II SFT. Of those receiving adjuvant RT, 31% received single-fraction Gamma Knife (GK) radiosurgery treatments, although the authors did not specifically compare the outcomes for GK versus conventional RT (1). Shin et al. performed a similar study in 2021, which showed resection to be superior to radiosurgery as an initial treatment. They showed no statistically significant benefit in OS or recurrence-free survival (RFS) for patients receiving adjuvant RT versus resection alone, although most of these patients received hyperfractionated regimens rather than radiosurgery (9). Since 2016 there has been scant evidence evaluating multi-fraction radiosurgery’s utility in treating SFT, although the available results have been encouraging. One 2021 report described an 87-year-old woman who received fractionated GK radiosurgery for SFT of the sella turcica that recurred after partial resection. After completion of GK, the patient’s visual field deficits resolved and she has been without recurrence for 15 months (11).

Stereotactic radiosurgery (SRS), specifically GK, has emerged as a promising post-operative treatment modality for SFT in the past decade. Multiple case series have demonstrated a likely survival benefit of adjuvant SRS compared to resection alone, with 3-year progression-free survival (PFS) of SFT treated with surgery and adjuvant SRS ranging from 60-92% in the literature (**Table 1**) (5–7). However, even with this PFS benefit, local recurrence after adjuvant radiosurgery remains prevalent (5, 6). Therefore, a need for more effective post-surgical treatment methods remains. Some studies, such as the 20-patient series by Kano et al. and the multi-center study by Cohen-Inbar et al., have found that higher cumulative radiation dose from adjuvant GK correlates with improved PFS for treatment of SFT (6–8).

**Table 1:** Previous studies on post-operative radiosurgery for recurrent or residual hemangiopericytoma.

Investigator, year	Patients (n)	Total tumors (n)	Margin dose (Gy)	Median target volume (mL)	Median follow-up (mos)	Tumor control at last FU	5 year OS (%)	1 year PFS (%)	3 year PFS (%)
Kano, 2008 (6)	20	29	15	4.5	37.9	72.4	85.9	89	66.7
Kim, 2010 (16)	9	17	18.1	2.2	33.8	82.4	NA	100	NA
Olson, 2010 (5)	21	28	17	4.6	68	46.4	81	60	60.3
Veeravagu, 2011 (15)	14	24	21.2	9.2	37	81.8	81	95	71.5
Tsugawa, 2014 (7)	7	10	16.5	4.1	52.1	70	85.7	100	92
Cohen-Inbar 2017 (8)	90	133	15	4.9	59	55	82	92	70
Kim, 2017 (14)	18	40	NA	NA	134.7	80	85.6	NA	NA

NA, Not available.

However, increasing cumulative dose in a single GK fraction to intracranial targets can be problematic, as it confers a greater risk of brain radiation necrosis, especially for larger treatment volumes (15). Increasing total GK dose by spreading treatment over multiple fractions is often avoided as it requires multiple stereotactic head frame placements for most systems. Head frame placement procedures are invasive and involve surgically securing an aluminum stereotactic head frame to the calvarium using screws or pins, which serves to enhance precision of radiation delivery. Our experience was unique in that we pursued an unconventional post-operative radiotherapy course consisting of a single-fraction GK treatment followed by a multi-fraction radiosurgery consolidation treatment with a linear accelerator. The GK treatment was delivered to four treatment volumes that included the entire resection cavity and three discrete tumor nodules at the cavity margins. The consolidation treatment was delivered to the entire resection cavity, and the enhancing tumor nodules were contoured. In total, the wide post-operative field received a cumulative dose of 20 Gy, while each radiographically high-risk area noted on initial MRI received 34-36 Gy. We were thus able to deliver a concentrated dose to high-risk areas that was significantly greater than the mean dose of 17-20 Gy described in most published case series of SRS-treated SFT (5–7). We achieved this while reducing the burden of invasive procedures for the patient, as stereotactic head frame placement was required for only one treatment session.

It is important to note that our experience occurred prior to the inception of the recently developed GK Icon™ and eXtend™ technologies, which do not require head frame placement and thus allow for multi-fraction treatment regimens. The GK Icon™ system allows for headframe and mask-based immobilization during treatment while the eXtend™ system uses a vacuum-sealed dental bite-tray and electronic probes to maintain inter and intra-fraction patient positioning (15, 16). These recently developed iterations of GK treatment are advantageous over our described treatment technique in that they do not require the interfraction transfer of treatment data from one system to another. Additionally, each of these systems incorporates cutting edge intrafraction motion management and interfraction repositioning systems, which allow for submillimeter targeting accuracy that cannot be achieved with linear accelerator-based treatment (15, 16). However, our described technique has some benefits over these systems. For example, because of the bite-tray based immobilization in the eXtend™ system, poor dentition or hypersensitive gag reflex can be barriers to treatment. It is also broadly thought that, despite the impressive precision of mask and bite tray-based systems, they are still not as precise as rigid, frame-based fixation (15). Additionally, most institutions with GK still only have access to headframe-based Perfexion™ systems, making our combination technique that incorporates this older system clinically relevant due to wider availability.

This case report is limited by a relatively short follow-up time, and further serial imaging and follow-up for this patient are necessary to more accurately assess this treatment method’s long-term efficacy. However, the lack of disease recurrence and the patient’s high quality of life several years post-treatment are very encouraging. We also believe our experience is a valuable contribution to the literature given the paucity of data describing treatment options for SFT as defined by the 2016 WHO guidelines. We hope that our reporting of this new radiosurgery paradigm may better inform the management of this uncommon and aggressive cancer.

## Conclusions

Post-resection Gamma Knife radiosurgery with multiple treatment volumes to target radiographically high-risk areas, followed by multi-fraction radiosurgery using a linear accelerator, could be a safe and effective way to treat recurrent, grade II SFT without significantly compromising patient quality of life.

## Data Availability Statement

The original contributions presented in the study are included in the article/supplementary material. Further inquiries can be directed to the corresponding author.

## Ethics Statement

Written informed consent was obtained from the individual(s) for the publication of any potentially identifiable images or data included in this article.

## Author Contributions

AA and DL contributed to the conceptualization, design, and writing of the original draft of this report. KR, JS, and CK contributed to the conceptualization and design of this report. All authors contributed to the reviewing and editing of this report. All authors contributed to the article and approved the submitted version.

## Conflict of Interest

The authors declare that the research was conducted in the absence of any commercial or financial relationships that could be construed as a potential conflict of interest.

## Publisher’s Note

All claims expressed in this article are solely those of the authors and do not necessarily represent those of their affiliated organizations, or those of the publisher, the editors and the reviewers. Any product that may be evaluated in this article, or claim that may be made by its manufacturer, is not guaranteed or endorsed by the publisher.
